# Coaching legacies: influence propagation through temporal social networks in the Australian Football League

**DOI:** 10.3389/fspor.2023.1172264

**Published:** 2023-07-25

**Authors:** Gordana Marmulla, Geoff Dickson, Hagen Wäsche, Ulrik Brandes

**Affiliations:** ^1^ Social Networks Lab, Department of Humanities, Social and Political Sciences, ETH Zürich, Zurich, Switzerland; ^2^ Department of Management and Marketing, La Trobe University, Melbourne, Australia; ^3^ Institute of Sports and Sports Science, Karlsruhe Institute of Technology, Karlsruhe, Germany

**Keywords:** social influence, temporal network, network analysis, influence propagation, sports network, coaching tree

## Abstract

We study the lineage network of coaches in the Australian Football League (AFL) using a novel process of influence propagation through temporal social networks. Coaching and being coached are considered major opportunities for learning, and the vast majority of AFL coaches are former AFL players. We, therefore, establish influence via two antagonistic components: as players, future coaches are influenced by their coaches, and later liberate themselves from these influences while being coaches themselves. Influence thus propagates through time-dependent player–coach relationships, and we obtain a ranking of coaches by their aggregated influence on others. In addition to being based on an explicit process, we argue that the ranking has face validity, because it indeed favors highly reputed coaches, and is not determined by temporal or activity indicators such as the starting year of a coaching career, its length, or the number of future coaches coached.

## 1. Introduction

We study influential coaches in Australian rules football through the lens of an evolving network of coaching relationships. Player–coach affiliation networks are among several kinds of social networks that are found increasingly valuable for addressing research questions in sports (1). Specifically, informal and personal experiences such as having been an athlete, being mentored, or observing coaches have been suggested to be even more important for coach development than formal education in courses or seminars (2–5).

As a potential source of influence on a coach’s views, approaches, and behaviors, we consider selected dyadic relationships that are expected to facilitate his or her development. Genealogy in relation to coaching was exemplified by Maxwell (6) in his popular book *The 21 irrefutable laws of leadership*. He observed that at the turn of the century, mentoring relationships between head and assistant coaches linked half of the coaches in the United States National Football League (NFL) directly or indirectly to just two historical figures. This observation was used to illustrate the *Law of Reproduction*: “It takes a leader to raise up a leader.” (6, p. 134). Not surprisingly, mentoring networks, or *coaching trees*, have evolved into a frequent discussion point in media reporting on the NFL.^1^ Coaching trees describe a coach’s sporting lineage in terms of coaching ancestors and descendants. They are often relied upon for recruitment, since a primary strategy for choosing a coach out of candidates is to examine with whom he has worked in the past and whether his mentors were successful coaches themselves (7). Coaching trees also provide an opportunity to analyze a coach’s influence on his assistant coaches (7) and can give insights into coach mentoring (8).

Similarly, networks of coaches and players who subsequently become coaches themselves can be constructed. The relevance of such coach–future coach coaching networks is suggested by studies on coaching development that emphasize a coach’s own playing experience (9–15). Besides its relevance in coaches’ development, the coach–athlete relationship was found to influence success (16), achievement of mastery goals (17), leadership behavior of athletes (18), group cohesion (19), developmental experiences of adolescents (20), athletes’ general happiness (21), and coach’s wellbeing (22), to name a few.

While practicing a sport, athletes experience and witness coaching methods, which are subsequently embodied and internalized (3). An embodied coaching culture might also be paraphrased as an individual’s “coaching philosophy,” the cumulative aggregation and reshaping of practices experienced as a player and coach. Following Bourdieu, a coaching philosophy could also be considered a coaching-specific *habitus*, which is “the active presence of the whole past of which it is the product” (23, p. 56). Lyle (24) views a coaching philosophy as a set of beliefs and behaviors that shape the individual coaching practice and reflect deeper values. Williams and MacNamara (25) found that coaches acknowledge the influence of former coaches on current coaching practices and beliefs, which they used as the foundation for their own personal development as a coach. Both previous and ongoing experiences are relevant for developing a coaching practice (26). Research suggests that coaching philosophies are already developed throughout playing careers and constantly adapted while coaching (27), even more, they are lifelong in development (28).

The present study examines the coaching network between AFL coaches and those of their players who become AFL coaches themselves. Almost all (95%) coaches in the AFL have played in the league prior to their coaching careers. Hence, the boundary of the lineage network of AFL coaches is comparatively well defined (29). Since records objectively document coaching relationships, the network is an almost complete and reliable proxy for conduits of influence.

We investigate the influence within the coaching network by applying a new model for social influence propagation. Mathematical models of social influence (30, 31) derive changes (e.g., behavioral changes) in individuals’ states as a consequence of their social interactions. In influence networks, nodes represent individuals and (directed) edges represent (directed) influence. As individuals can act as both the source and target of influence, influence is enabled to propagate through the network. Most of the research literature on influence propagation models is on *static* social networks (32), where a dynamic influence process on a *static* influence network is modeled. The new model extends previous models to accommodate changes in the underlying network over time, representing a dynamic influence process on a *temporal* influence network.

Our proposed model of influence propagation over temporal networks establishes influence relationships between any two coaches by two antagonistic components: the susceptibility of players to be influenced by their coach(es) and growing independent from prior influences through coaching experiences. We construct a hypothetically directed, weighted, temporal network of influence using the *formal* coaching relationship as a proxy. Thus, playing under a coach equals being influenced by him. The degree of influence associated with an edge is the annual proportion of matches in which a coach coached a future coach, i.e., the more often a player is exposed to a coach’s coaching, the higher the influence. Also, the higher the general susceptibility to influence, the greater the degree of transmission. We construct individual temporal weighted independence using the *formal* coaching activity as a proxy. Thus, coaching equals growing independent from the influence received when playing under other coaches. The degree of independence is associated with the annual proportion of matches a coach has coached. From the influence relationships established by the process, we identify the most influential coaches in AFL history by summing up their influence on others.

The remainder of this paper is organized as follows. In Section 2, we examine the properties of coaches, players, and their formal coaching relationship, and we embed them within the Australian Rules Football environment and its professional league, the AFL. Our novel process of influence propagation is outlined in Section 3, followed by its application to AFL coaches. Results and limitations are discussed in Section 4. We conclude with suggestions for future research in Section 5.

## 2. Background and data

In this section, we focus on common features of AFL coaches, AFL players who become coaches, and their relationship to each other. The aim is to provide a contextual background for assessing our proposed influence propagation process.

### 2.1. Terminology

With the term “coach” we refer to a club’s head coach. Players who become coaches at some point are labeled future coaches. For comparison, players who do not become coaches are still considered. These players are referred to as future non-coaches. The term “players” refers to all players, independent of whether they will or will not become coaches. Players who simultaneously played and coached a match at least once are referred to as player-coaches. For context, the club for which coaches and players participate in the matches is also included.

### 2.2. Data

We obtained all data from the AFL Tables website,^2^ which in turn sources its data from books by Rodgers and Browne (33), Blair (34), Holmesby and Main (35), newspapers and magazines, and the official website of the AFL.^3^ The website is a private project and has no official association with the AFL. After following internal checks and sample validity, and given the 20-year history and broad community support, we nevertheless assume that the records are sufficiently complete and accurate to demonstrate the viability of our approach.

We compiled lists of player and coach names for each game in the AFL (formerly known as Victorian Football League, VFL) since 1897 who played or coached in a match. This compilation excludes players who never played but were nevertheless on a club’s roster. We argue that this exclusion is not a substantial limitation because most of the players named on a club’s roster also played at least once per season. The team sheet will list 23 players for any match, one of whom acts as a medical substitute, and no more than 18 players are permitted to be on the field at any moment. The mean value of the average number of competing players per club over an entire season is 35.6. This value is close to the minimum roster size, which has always been approximately 50 before the early 1990s and approximately 40 since then. The lineage network of coaches was established by matching player and coach names and identifying future coaches’ coaches for each season.

### 2.3. Monadic attributes

We give a few summary descriptives about the league, its coaches, and players individually, before considering relationships among them.

*The AFL is a growing and (nearly) closed system.* From 1897, the number of clubs participating in the AFL increased steadily, except for 1916 due to World War I. The growing number of participating clubs resulted in an increased number of matches, coaches, and players. Initially, the number of coaches was often smaller than the number of participating clubs, but from 1925 onward,, it was consistently larger. This difference shows that not every team had a coach unique to that role in the league’s early days, whereas later teams would employ multiple coaches in a season. Until the end of the 2020 season, 12,848 players, 365 coaches, and 21 clubs had been associated with the AFL. However, less than 3% of players (347 out of 12,848, or 2.7%) subsequently became AFL coaches. We also note that 347 of the 365 coaches, or 95%, played at least one game in the AFL.

*Playing and coaching careers are only getting slightly longer.* The slight increase in the length of playing and coaching careers is evidenced by weak positive Kendall’s rank correlation coefficients $\tau_{B}$ between the season of the first match coached or played and the number of seasons a coach or player is active in the respective capacity. Growth is the largest for playing careers of future non-coaches ($\tau_{B}=0.16$, p<0.001), followed by coaching careers ($\tau_{B}=0.11$, p<0.001), and the smallest for future coaches ($\tau_{B}=0.09$, p<0.05). Currently active players and coaches have been excluded from this analysis to prevent any bias.

*On average, future coaches are competing in more than three times as many matches compared to future non-coaches.* The difference becomes even more pronounced when comparing the medians (see Table 1). The higher number of matches played by future coaches has three reasons. First, future coaches play on average more than twice as many seasons as future non-coaches. Second, future coaches are part of more successful teams, competing on average in more than four times as many finals series matches as future non-coaches. Third, future coaches play more regularly, being selected for an average of 70% of their club’s matches per season compared to only 38% for future non-coaches.

**Table 1:** Main statistics of the distribution of the total number of matches, the number of matches part of the final series, and the number of seasons for coaches and future (non-)coaches.

		Min	Q1	Median	Mean	SD	Q3	Max
Coach	Matches	1	17	44	81.9	115.0	91	718
	Final series	0	0	0	3.5	8.1	3	58
	Seasons	1	1	2	4.4	5.3	5	38
Future non-coach	Matches	1	5	18	46.5	62.5	65	432
	Final series	0	0	0	1.9	4.1	2	39
	Seasons	1	1	3	4.1	3.6	6	23
Future coach	Matches	1	106	154	158.7	76.2	206	403
	Final series	0	2	8	8.8	7.2	14	29
	Seasons	1	8	10	10.4	3.9	13	20

The statistics consist of the minimum (min), first quartile (Q1), median, mean with standard deviation (SD), maximum (max), and third quartile (Q3).

**Figure 1:**
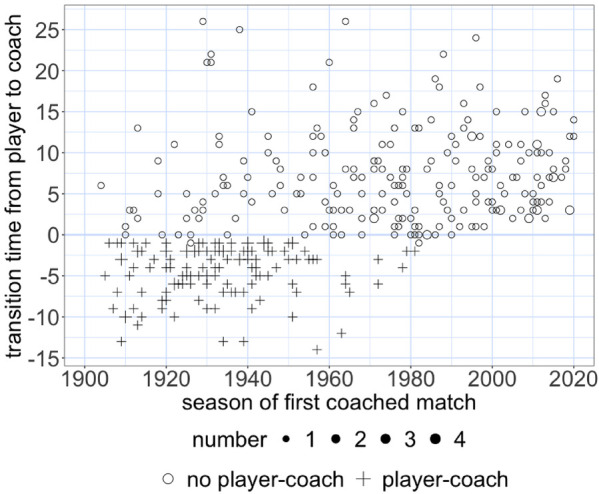
The time to transition from player to coach over time. The horizontal zero line represents a smooth transition where the season of the first coached match follows the one of the last played.

*Over time in the AFL, the transition from player to coach takes marginally longer for non-player-coaches, and the player-coaches disappear.* We define the transition duration from player to coach as the number of seasons between a player’s last game and his first game as a coach. For non-player-coaches, a minor prolongation is evidenced by a weak positive rank correlation coefficient between the season of the first coached match and the transition duration ($\tau_{B}=0.09$, p<0.1). Figure 1 shows the coaches’ positions relative to the horizontal zero line that represents a smooth (the first coached match is in the subsequent season of the last played one) transition from player to coach. In the AFL’s early years, coaches are often below the zero line because they were player-coaches or coaches during their playing career out of necessity. Player-coaches account for 35% of all coaches. Around 1980, both the player-coaches and coaches who coached their first match before playing their last disappeared. The media recently speculated whether player-coaches might come back due to financial issues induced by COVID-19.^4^ However, this did not transpire.

*Successful coaches have longer careers.* As unsuccessful coaches are likely to have shorter tenures, a relationship between career length and success is expected. For this investigation, we set the career length to a weighted career span (i.e., the cumulative proportions of coached matches relative to the possible maximum of the associated club). There are alternatives to weighted career span, e.g., the absolute number of matches or seasons coached. However, the alternatives might bias the relationship. In the case of matches, a bias is created by the increased number of matches induced by the league’s growth. Whereas in the case of seasons, no difference would be made between coaches who have coached one or all matches of a season. There is evidence for a monotone relationship given the positive correlation coefficient between weighted career span and the number of premierships won by coaches ($\tau_{B}=0.42$, p<0.001). The association is weaker between the weighted span and the average rank in the ladder before the final series ($\tau_{B}=-0.13$, p<0.001). This difference in the correlation coefficient’s strength indicates that the final series’ performance is a decisive factor for coaches’ continued employment and, thus, their career lengths.

### 2.4. Dyadic attributes

We now turn to the properties of dyadic relationships between coaches, players, and clubs regarding coaching.

*Coaches and future coaches have more joint seasons than coaches and future non-coaches.* We denote the average number of joint seasons with $s_{{}_{\emptyset }}$. From the perspective of players, future coaches have, on average, around one more joint season with a coach than future non-coaches ($s_{{}_{\emptyset }}=3.2$ vs. $s_{{}_{\emptyset }}=2.0$ seasons, see Table 2). From the perspective of coaches, the average number of joint season with future coaches and future non-coaches is more similar in duration ($s_{{}_{\emptyset }}=2.4$ vs. $s_{{}_{\emptyset }}=2.1$ seasons, see Table 2).

**Table 2:** Main statistics for coaches and future (non-)coaches regarding the distribution of the umber of dyadic associates (#) and the average number of seasons a relationship was present in ($s_{{}_{\emptyset }}$).

		With	Min	Q1	Median	Mean	SD	Q3	Max
Coach	#	Future coach	0.0	1.0	3.0	3.7	3.9	5.0	26.0
		Future non-coach	12.0	33.0	53.0	70.6	60.3	81.0	339.0
	$s_{{}_{\emptyset }}$	Future coach	1.0	1.0	1.9	2.4	1.8	3.0	11.2
		Future non-coach	1.0	1.0	1.7	2.1	1.3	2.6	6.7
Future coach	#	Coach	0.0	2.0	3.0	3.8	2.3	5	14.0
	$s_{{}_{\emptyset }}$	Coach	1.0	1.9	2.7	3.2	2.1	3.8	16.0
Future non-coach	#	Coach	0.0	1.0	2.0	2.1	1.6	3.0	12.0
	$s_{{}_{\emptyset }}$	Coach	1.0	1.0	1.5	2.0	1.3	2.3	14.0

**Figure 2:**
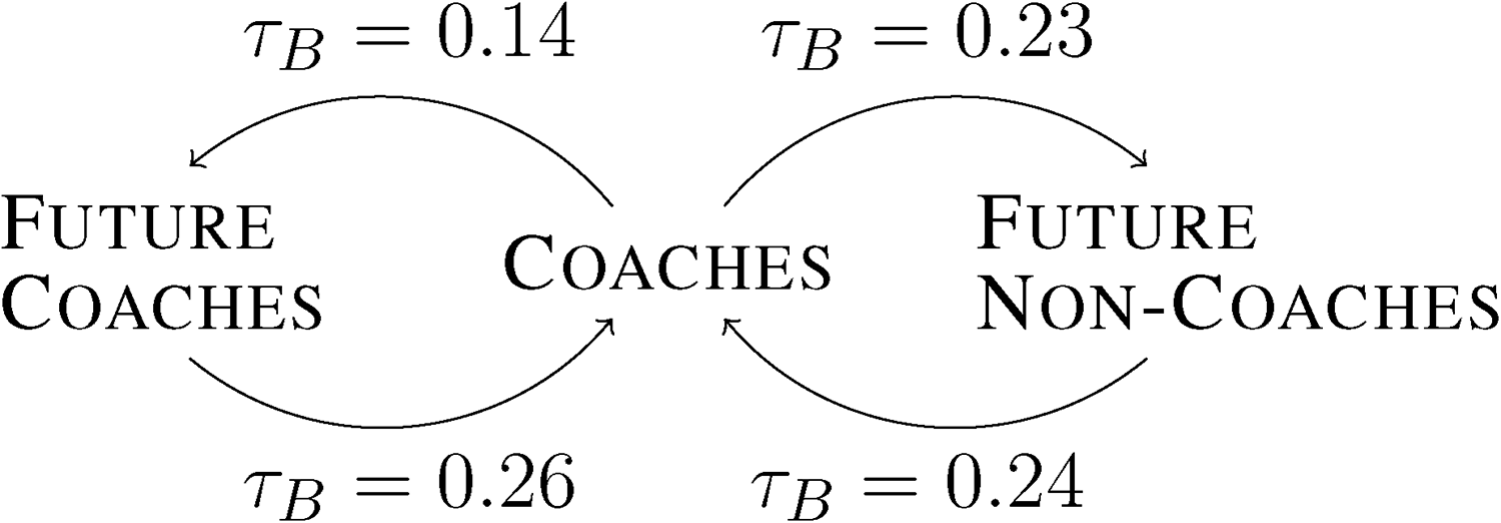
Kendall’s rank correlation coefficients $\tau_{B}$ between the season of the first coached match of a coach and the average number of joint seasons with a future (non-)coach, as well as between the season of the first played match of a future (non-)coach and the average number of joint seasons with a coach; all p<0.001.

*Over time, coaches and players have more joint seasons.* This tendency is indicated by positive correlation coefficients $\tau_{B}$ between $s_{{}_{\emptyset }}$ and the season of the first match played or coached by the corresponding future (non-)coach or coach (see Figure 2). Currently active players and coaches were excluded from all correlations $\tau_{B}$ to prevent bias. There are two reasons for the increase in the number of joint seasons. First, the playing and coaching careers are getting slightly longer in the AFL. A prolonged career reduces accessible positions for newcomers, as there are upper bounds for both associated coaches and players. As a result, the average number of first-time players and coaches per club decreases over the seasons. The decrease is indicated by negative correlation coefficients $\tau_{B}$ between the season and the average number of first-time coaches and future (non-)coaches per club. It is the largest for future non-coaches ($\tau_{B}=-0.68$, p<0.001), followed by the future coaches ($\tau_{B}=-0.29$, p<0.001) and the smallest for the coaches ($\tau_{B}=-0.24$, p<0.001). Second, there is no evidence of increasing relocation of coaches and future (non-)coaches. We refer to a relocated coach or player if they were on the team sheet of a club other than the club of their last match. The lack of evidence is indicated by non-significant correlation coefficients between the season and the average number of relocated coaches and future (non-)coaches per club. For players, the steady level of relocation might be supported by the AFL’s salary cap that prevents increasingly profitable transfers between clubs season after season.

*The relative offspring of coaches decreases over time.* We define a coach’s relative offspring as the number of coached future coaches relative to his number of coached seasons. The correlation between the season of a coach’s first match and his relative offspring indicates a negative association ($\tau_{B}=-0.33$, p<0.001). Coaches who are still active, or coached players who are still active, were excluded as they can bias the relationship. The negative association is the result of two related factors. First, the number of new future coaches decreases over time. Second, coaches’ tenures in the AFL are growing longer over time. Both factors serve to reduce a coach’s relative offspring.

## 3. Influence propagation

The proposed influence propagation model assumes that influence relationships between subjects evolve over time through two antagonistic components: the susceptibility to influence from coaches while a player and growing independent from past influence of others through experience gathered as a coach. They are explained in more detail below.

**Figure 3:**
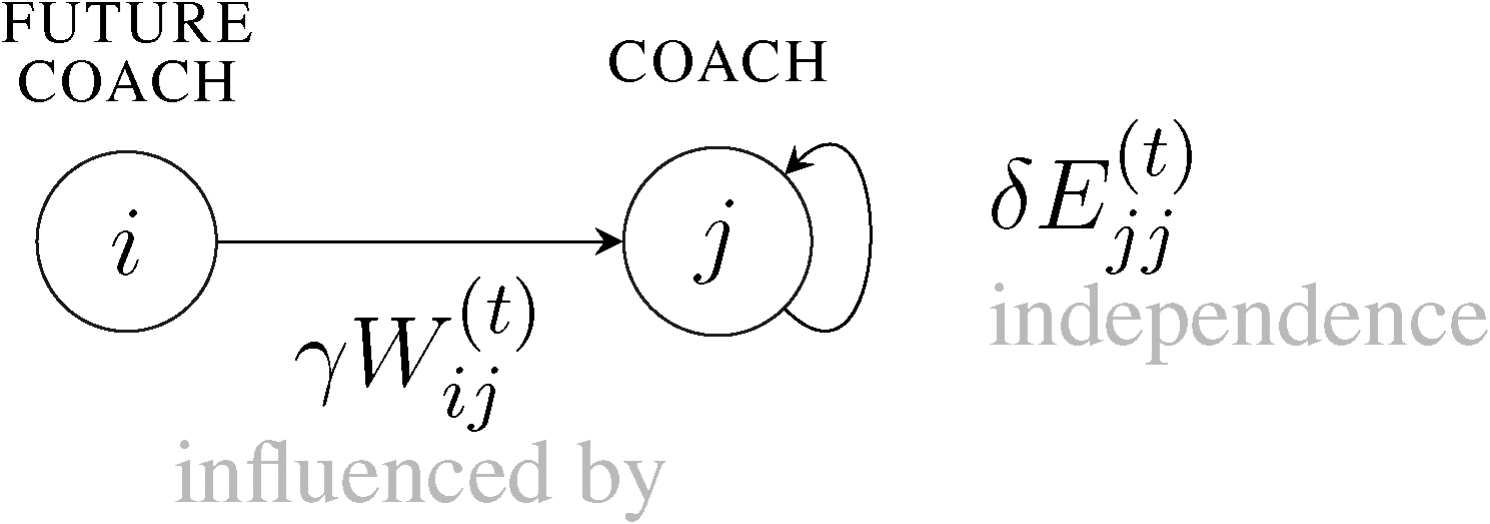
A visualization of the components from the network perspective.

On the macro level, the extent to which these components apply is controlled by two parameters. Influence is controlled by a parameter γ ranging from zero (i.e., no interpersonal influence) to one (i.e., total influence). Independence is controlled by a parameter δ ranging from 0 (no independence) to 1 (total independence). On the micro level, influence and independence are adjusted for exposure time and indirect success, respectively. An example visualization of the components in the network perspective is shown in Figure 3. At any moment, every coach (i.e., a node in the coaching network) has a state that reflects his influence relationships. The influence relationships are expressed in a distribution. A node’s distribution describes the proportion of influence received by others or itself. We use the term self-influence to refer to the share of influence on oneself, i.e., the accumulated independence.

### 3.1. Influence

Influence is modeled as an assimilative influence. The influence relationships of a future coach become more similar to those of his coach after being coached by him. The future coach (i.e., the target of influence) gets influenced directly by the coach (i.e., the source of influence) and indirectly by all of the coach’s influencers. This assimilation is implemented as a *weighted average* of both influence relationships (i.e., distributions). The extent of assimilation is regulated by the product of the general susceptibility parameter γ and a time-dependent dyadic influence weight $W_{ij}^{\left(t\right)}$. As a *proxy*, the time-dependent dyadic influence weight is set to the proportion of the future coach’s matches in which he played under the coach relative to the total number of matches he has played throughout season t. An approximation is necessary since the influence cannot be measured directly.

### 3.2. Independence

Independence is modeled as the decay of influence and simultaneous gain of self-influence. Coaches acquire independence by separating themselves from their prior influences and thus developing their own coaching philosophies. The separation is realized by multiplying the coach’s distribution with a scaling factor smaller than 1. The simultaneous development of a personal coaching philosophy is realized through an additive increase of the self-influence by the amount lost by the scaling. In this way, separation and development inherently depend on each other. The extent is regulated by the product of the general independence parameter δ and a time-dependent local independence weight. As a *proxy*, the time-dependent independence weight is set to the proportion of a coach’s coached matches relative to his associated club’s total number of matches. This proportion implicitly makes the weight depend on a coach’s success because it is not uncommon for an unsuccessful coach to be replaced in the middle of a season. However, the weight is not explicitly dependent on success because all matches are treated equally, as no distinction is made between regular matches and those from the final series.

### 3.3. Formalization

The novel process of influence propagation can be expressed as a recursive formula of operations on directed and weighted adjacency matrices:

(1) $$I^{\left(t\right)}=\underset{\begin{array}{c} \text{decay\textbackslash{} of}\\ \text{ influence} \end{array}}{\underbrace{\left[I_{id}-\delta E^{\left(t\right)}\right]}}\underset{\begin{array}{c} \text{influence}\\ \text{ propagation} \end{array}}{\underbrace{[\gamma W^{\left(t\right)}+(1-\gamma )I_{id}]I^{\left(t-1\right)}}}+\underset{\begin{array}{c} \text{growing }\\ \text{independent} \end{array}}{\underbrace{\delta E^{\left(t\right)}}}$$

with row stochastic $I^{\left(0\right)},W^{\left(t\right)}\in [0,1{]}^{n\times n}$, diagonal $E^{\left(t\right)}\in [0,1{]}^{n\times n}$, $I_{id}$ as identity matrix of appropriate size, and parameters γ,δ∈[0,1]. A single entry $I_{ij}^{\left(t\right)}$ describes the proportion of influence coach j has on i after time step t. Therefore, the ith row $I_{i⋆}^{\left(t\right)}$ describes the influence distribution of coach i after time t. As a result, $I^{\left(t\right)}$ is row stochastic.

#### 3.3.1. Influence network

The hypothetical temporal influence network is captured in the matrix $W^{\left(t\right)}\in [0,1{]}^{n\times n}$. A single entry $W_{ij}^{\left(t\right)}$ is larger than 0 if coach j influences future coach i at time t. The extent to which a coach influences a future coach is described by the weight $W_{ij}^{\left(t\right)}\in [0,1]$, which is approximated by the proportion of their joint matches during season t. We defined matrix $W^{\left(t\right)}$ as

(2) $$W_{ij}^{\left(t\right)}=\left\{ \begin{array}{cc} \frac{M_{ij}^{\left(t\right)}}{\sum_{\;j=1}^{n}M_{ij}^{\left(t\right)}}, & \text{if}\;i\neq j\;\text{and}\;\sum_{\;j=1}^{n}M_{ij}^{\left(t\right)}\neq 0\\ \frac{M_{ii}^{\left(t\right)}}{\sum_{\;j=1}^{n}M_{ij}^{\left(t\right)}}, & \text{if}\;i=j\;\text{and}\;\sum_{\;j=1}^{n}M_{ij}^{\left(t\right)}\neq 0\\ 1, & \text{if}\;i=j\;\text{and}\;\sum_{\;j=1}^{n}M_{ij}^{\left(t\right)}=0\\ 0, & \text{otherwise} \end{array}\right.$$

where $M_{ij}^{\left(t\right)}$ denotes the number of games future coach i has played for coach j during season t. If multiple coaches are listed for a club in a match, the match’s count of “1” is uniformly split among the coaches. For example, with North Melbourne vs. Geelong (15th June 1940),^5^ two coaches are listed for North Melbourne. Therefore, for future coach Len Thomas, this match contributes to the number of joint matches with Wally Carter and Jim Adamson by 1/2 each. However, this situation is rather rare, as there are only 11 out of 15,776 matches in which a team has more than one coach. In entry $M_{ii}^{\left(t\right)}$, two kinds of matches are accumulated for node i: future coach i has competed either without a coach or as a player-coach in season t. The former occurred mainly in the early years of the AFL up to 1922. The latter occurs repeatedly. Player-coaches were present from 1905 to 1981.

The weight with which a coach influences a future coach was defined as a proportion for multiple reasons. First, an influence weight is the same for future coaches who played the same proportion under a coach regardless of how many matches they played in total. In this way, the assumption is realized that future coaches, who are part of a team the whole season but might not stand on the ground for every match due to performance reasons or injuries, are influenced to the same extent. Next, transfers of future coaches *during* a season can be handled easily, which occurred in nine (out of 156 total transfer) cases. Finally, the increasing density of play over time is compensated. In conclusion, the definition of $W^{\left(t\right)}$ implies that its largest possible dyadic influence weight is one and is achieved when a future coach has performed all of his matches under a single coach. By construction, $W^{\left(t\right)}$ is row stochastic.

The time-dependent dyadic influence weights together with the time-independent global susceptibility to influence γ are combined as

(3) $$P^{\left(t\right)}=\gamma W^{\left(t\right)}+(1-\gamma )I_{id}$$

The matrix $P^{\left(t\right)}$ consists of three non-zero entries:

(4) $$\begin{array}{rl} & P_{ij}^{\left(t\right)}=\\ & \left\{ \begin{array}{ccc} \gamma \frac{M_{ij}^{\left(t\right)}}{\sum_{\;j=1}^{n}M_{ij}^{\left(t\right)}}, & \text{if}\;i\neq j\text{ and }\sum_{\;j=1}^{n}M_{ij}^{\left(t\right)}\neq 0 & \\ (1-\gamma )\left[1+\frac{\gamma }{1-\gamma }\frac{M_{ii}^{\left(t\right)}}{\sum_{\;j=1}^{n}M_{ij}^{\left(t\right)}}\right], & \text{if}\;i=j\;\text{and}\;\sum_{\;j=1}^{n}M_{ij}^{\left(t\right)}\neq 0 & \\ 1, & \text{if}\;i=j\;\text{and}\;\sum_{\;j=1}^{n}M_{ij}^{\left(t\right)}=\;0 & \\ 0, & \text{otherwise} & \end{array}\right. \end{array}$$

that correspond to two different events. First, the coach’s (coaches’) influence and the future coach’s inertia toward this influence. Second, preservation entries ensure that a node’s influence distribution does not change if no influence has happened. The matrix $P^{\left(t\right)}$ is row stochastic since it is a weighted average of two row stochastic matrices $W^{\left(t\right)}$ and $I_{id}$.

#### 3.3.2. Independence network

The time-dependent individual rates for becoming independent are comprised in the diagonal matrix $E^{\left(t\right)}\in [0,1{]}^{n\times n}$. A single entry $E_{ii}^{\left(t\right)}$ is larger than 0 if coach i has coached in season t. The extent to which a coach becomes independent is described by the weight $E_{ii}^{\left(t\right)}$, which is approximated by the proportion of joint matches with the coach’s associated club. The matrix is defined as

(5) $$E_{ij}^{\left(t\right)}=\left\{ \begin{array}{cc} \sum_{c}\frac{C_{ic}^{\left(t\right)}}{g_{c}^{\left(t\right)}}, & \text{if}\;i=j\\ 0, & \text{otherwise} \end{array}\right.$$

with $C_{ic}^{\left(t\right)}$ as the number of matches that coach i has coached throughout the season t for club c, whereas club c has had $g_{c}^{\left(t\right)}$ games in total. Every coached match counts as a complete match for a coach, even in the rare case of more than one coach for the club. This counting induces that the extent of independence is not dependent on whether there has been a second coach or not.

For two reasons, the weight with which a coach becomes independent was defined as a proportion. First, the independence weight is the same for coaches who have coached the same proportion of their clubs’ matches. This makes the rate of independence not dependent on the performance of a club and the mode of play, as both let $g_{c}^{\left(t\right)}$ vary. Second, the increased match density induced by the league’s growth is compensated. In conclusion, the individual independence rate is the largest for a coach if he has coached *all* matches of his associated club during the season.

### 3.4. Initial independence and the unexplained

In general, the initial influence relationships could be chosen as any row stochastic matrix $I^{\left(t_{0}\right)}$. A straightforward choice is $I^{\left(t_{0}\right)}=I_{id}$, where every node is initially completely independent. However, the choice of $I^{\left(t_{0}\right)}=I_{id}$ lets the root nodes in the coaches’ lineage network occupy a special position. A root node is a coach who has never been influenced by another coach. Root nodes arise in the following two situations. First, the root node never played in the AFL. Second, the root node played, but the club had no coach, which occasionally occurred until 1922. Root nodes have a special position regarding the influence because of the following circumstance. The natural evolution of initially being a player and then a coach implies a decrease in self-influence as a player and an increase when becoming independent as a coach. However, the root nodes receive no influence before their coaching career. This lack of received influence implies for $I^{\left(t_{0}\right)}=I_{id}$ that they could directly spread their high level of self-influence from the first coaching season. Therefore, to ensure a fair and similar situation for the start of the coaching career among the coaches, the initial independence parameter $\delta_{0}$ was introduced. This initial independence $\delta_{0}$ describes the proportion of self-influence with which each node starts at $t_{0}$ and can range from 0 (completely dependent) to 1 (completely independent). The remaining $1-\delta_{0}$ amount of influence is attributed to an auxiliary node. The auxiliary node represents the network’s *unexplained* influence, of which is unknown where it comes from. Introducing the auxiliary node entails an opportunity to make the root nodes lose their special position by choosing a small $\delta_{0}$ which forces them to become independent before spreading a higher level of their influence. The added auxiliary node changes the matrices, which describe the influence propagation process. The square matrices are extended by one dimension, and the new entries are defined as follows. The initial relationships are

(6) $$I^{\left(t_{0}\right)}=\left(\begin{array}{cc} \delta_{0}\;I_{id} & 1-\delta_{0}\\ 0 & 1 \end{array}\right)$$

where bold letters represent vectors of the appropriate size. For any t, the influence and independence matrices are enlarged by

(7) $$\begin{array}{rl} P_{n+1\;i}^{\left(t\right)} & =P_{n+1\;i}^{\left(t\right)}=\left\{ \begin{array}{cc} 1, & i=n+1\\ 0, & i\neq n+1 \end{array}\right. \end{array}$$

(8) $$E_{n+1\;i}^{\left(t\right)}=E_{i\;n+1}^{\left(t\right)}=0\;\text{ for all}\;i.$$

Coaches propagate the unexplained influence if they have a non-zero proportion in their distribution from the auxiliary node when influencing others. As a result, the final amount of unexplained influence depends not only on the initial independence parameter $\delta_{0}$ but also on γ and δ.

### 3.5. Index choice for a ranking

The influence relationships after the last season of 2020 can be used to establish a ranking for the most influential coaches. The ranking is established by applying a centrality index to $I^{\left(2020\right)}$. Of various possibilities, here, the chosen centrality index is the weighted indegree excluding loops. This index computes the total influence of a node as the cumulative sum of all outgoing influence on others. In this way, homogeneity among the nodes is indirectly assumed, i.e., every node is equally important.

### 3.6. Impacts of parameters

The parameters γ,δ, and $\delta_{0}$ affect the influence relationships $I^{\left(t\right)}$. Different parameter choices are linked to different characteristics of the modeled influence propagation, which, in turn, favors different characteristics of coaches as being influential. The following links were found under the homogeneity assumption of the coaches.

First, for γ in isolation ($\delta_{0}=\delta =0$), a small susceptibility to influence makes the influence spread slowly and directly. The slowness favors coaches with long careers and stable relationships with future coaches. The directness favors coaches whose coaching lineages evolve into breadth rather than depth. On the contrary, a high level of susceptibility to influence makes the influence spread quickly and indirectly. The quickness favors coaches who have unstable relationships with future coaches, and these are often coaches with short careers. The indirectness favors coaches whose coaching lineages evolve into depth rather than breadth. Moreover, large γ favors coaches who have themselves little history as players.

Next, a low level of independence δ causes becoming independent to be slow. The slowness favors coaches with long careers. This slowness of becoming independent is accompanied by the persistence of influence. Persistence favors coaches who occupy a position toward the roots. On the contrary, a higher independence rate causes the influence to become more volatile. The volatility favors coaches, which occupy a position toward the leaves. The leaves of the network are coaches that have not coached any future coach.

Finally, a high level of initial independence $\delta_{0}$ favors root nodes, i.e., coaches who either never played under an AFL coach or never played in the AFL. A small initial independence neutralizes this advantage.

### 3.7. Choice of parameters

We based our choice of δ on the study of Mallett et al. (13) and derived the other parameter values under some considerations. In this study, participating coaches reported that their experiences as an athlete are mostly a valued source in the first and middle but less in the last 2 years of their coaching career. The participants had, on average, around 17 years of coaching experience. We combined their reports with their average years of coaching. As a result, we set δ=0.1, such that a coach with the mean career span would be mostly independent of others’ influence again until his final 2 coaching years, even when he started his coaching career with no self-influence. From δ=0.1, we derived γ from the perspective of making experiences. Influence and independence can also be seen as two types of experiences made once while being a player and once while being a coach. The extent to which both types of experiences leave a mark was assumed to be the same. Therefore, γ was set to γ=δ=0.1. The third parameter, the initial independence $\delta_{0}$, was chosen small to avoid the root nodes occupying a special position. Nevertheless, $\delta_{0}$ was chosen as larger than 0, such that a root node would not just propagate unexplained influence in its first season. Therefore, the initial independence was set to $\delta_{0}=\delta =0.1$, corresponding to root nodes that would have already coached for one season.

### 3.8. Results

The total influence values for the top 20 coaches are presented in Table 3. The values result from the propagation and aggregation under the chosen parameters and centrality index. The influence values in Table 3 are augmented with some statistics on the coaches’ careers.

**Table 3:** Ranking of the top 20 influential coaches resulting from the proposed process of influence propagation together with some statistics of the coaches’ success and coaching relationships.

Rank	Coach	Influence	Begin of coaching	Hall of Fame^6^ (year of induction)	Premierships	Player-coach	#Seasons	#Players	#Future coaches	$s_{{}_{\emptyset }}$Player	$s_{{}_{\emptyset }}$Future coach
1	Jock McHale	9.17	1912	Legends (2005)	8	x	38	361	22	3.4	7.1
2	Allan Jeans	4.28	1961	Coaches (1996)	4		26	266	18	3.2	6.6
3	Norm Smith	4.00	1949	Legends (2007)	6	x	23	290	14	2.8	5.6
4	Dan Minogue	3.92	1920	Players (1996)	2	x	20	365	26	1.9	2.7
5	Frank Hughes	3.29	1927	Coaches (1996)	5		20	256	18	2.6	3.8
6	Kevin Sheedy	3.14	1981	Coaches (2008) Legends (2018)	4		29	279	12	3.9	8.7
7	Tom Hafey	3.11	1966	Coaches (1996)	4		23	336	20	2.6	5.0
8	Ron Barassi	2.82	1964	Legends (1996)	4	x	24	349	18	2.5	4.1
9	Dick Reynolds	2.66	1939	Legends (1996)	4	x	22	209	11	3.4	6.8
10	Reg Hickey	2.20	1932	Players (1996)	3	x	17	214	13	2.6	4.7
11	John Worrall	2.03	1902	Players (1996) Coaches (1996)	5		16	257	9	2.1	4.6
12	John Kennedy	1.83	1957	Legends (2020)	3	x	20	248	13	2.8	4.9
13	Perce Bentley	1.65	1934	Players (1996)	3	x	22	244	9	2.9	4.6
14	Leigh Matthews	1.32	1986	Legends (1996)	4		20	219	10	3.3	5.7
15	Mick Malthouse	1.24	1984	Coaches (2019)	3		31	346	9	3.2	5.6
16	Denis Pagan	1.08	1993		2		15	170	9	3.1	5.2
17	Phonse Kyne	1.07	1950	Players (1996)	2	x	14	158	7	3.2	5.9
18	David Parkin	1.04	1977	Coaches (2002)	4		22	286	16	2.8	3.6
19	Norman Clark	0.97	1912		2	x	13	254	17	1.7	1.9
20	Jack Hale	0.84	1948		0		10	151	6	2.4	4.3
	Average coach	0.21			0.3		4.4	74.2	3.7	1.7	1.8
	Unexplained	180.18									

The symbol # refers to the number of, and $s_{{}_{\emptyset }}$ refers to the average number of seasons of coaching relationships.

## 4. Discussion

By far, *Jock McHale* is the most influential coach. From the second rank on, the difference in influence between the coaches becomes smaller. Nevertheless, all of them stand out in comparison to the influence of the average coach. The total amount of influence in the network equals the number of nodes, i.e., the number of coaches (365) in addition to the auxiliary node. With an amount of 180.18, around half of the coaches’ total influence remains unexplained.

The robustness of the results was investigated with correlation tests for parameter values that ranged around the selected values. Rank^7^ correlation tests with Kendall’s $\tau_{B}$ have shown for varying all three parameters independently of each other with values of 0.02×x for $x\in N_{>0}$ and x≤10 that all rankings are significantly (all p<0.001) correlated. The minimum correlation coefficient of 0.75 arises for the parameter combinations $\gamma =\delta_{0}=0.02,\delta =0.2$, and $\gamma^{\prime }=\delta_{0}^{\prime }=0.2,\delta^{\prime }=0.02$. Two conclusions can be drawn from the rank correlations. First, the rankings do not change dramatically if the parameters vary slightly. Second, the rankings differ the most if the model’s antagonistic components are of opposite strength, i.e., if either influence or independence is predominant while the other is barely present.

Four notable observations about the characteristics of the top 20 most influential coaches exist. First, almost all the top twenty coaches have been inducted into the AFL’s Hall of Fame. Moreover, several top 20 coaches hold the *Legend* status that individuals receive “if they have had a particularly significant positive impact on the game of Australian Football.”^8^ The number of Legends is capped at 10% of the total Hall of Fame size, which currently results in 29 Legends.^9^ Of the 29 Legends, around 25% made it into the top 20 most influential coaches, according to the model. Second, namesakes of some of the AFL’s medals and trophies are among the top 20 most influential coaches:

∙ *Jock McHale Medal* for the coach of the AFL premiership’s winning team,
∙ *Norm Smith Medal* for the best player on the ground in the Grand Final of the AFL, and
∙ *Leigh Matthews Trophy* for the Most Valuable Player in the AFL of the season.

Third, almost any top 20 coach has won the premiership multiple times. Coaches winning premierships are more likely to coach for a longer time. Long-time coaches potentially coach more future coaches. Coaching more future coaches offers the opportunity to spread influence. Therefore, the co-occurrence of influence and success is not surprising. However, it is highlighted that the model does not explicitly consider the success of a coach. Fourth, 50% of the top 20 coaches were player-coaches. With this 50%, the player-coaches are over-represented among the most influential coaches, as their share among all coaches is only 35%.

**Figure 4:**
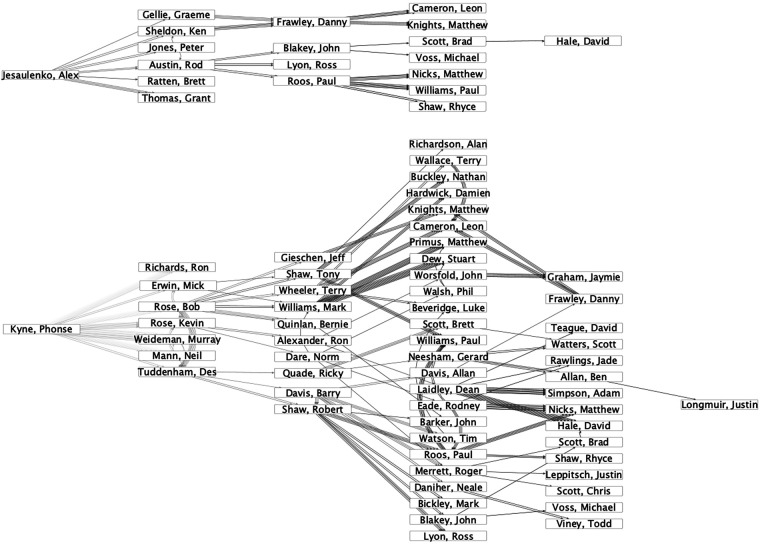
Network of coaching relationships (source node has coached target node) starting at a more (Phonse Kyne) and less (Alex Jesaulenko) influential coach; positioning of the coaches in x-direction corresponds to the minimal number of hops necessary to reach the coach from the focal nodes, the color of the links represents the season ranging from 1950 (lighter) to 2010 (darker).

There are also highly successful coaches who are not especially influential coaches. Two examples with different reasons are presented. First, *Alastair Clarkson* is ranked 48th with a total outgoing influence value of 0.29. However, he is a very successful coach who has already won four premierships during 16 seasons. Most other coaches needed more than 20 seasons to win four premierships. His lack of influence is because, as a recent coach, his coaching lineage has not had time to evolve. His former players may become coaches themselves in years to come, increasing his influence. Second, *Alex Jesaulenko* is ranked 102nd with a total outgoing influence value of 0.08. His lack of influence is a result of a sparse coaching lineage. In Figure 4, his coaching lineage is compared to *Phonse Kyne’s* (ranked 17th). The figure reveals that Alex Jesaulenko has about as many direct descendants as Phonse Kyne. Nevertheless, his influence propagation dissolves while the one of Phonse Kyne continues to spread.

Inspecting the top 20 coaches, we find that the ranking does not reduce to an ordering along a single characteristic, i.e., influence is not monotonically dependent on a straightforward attribute. The most influential coaches neither have the highest number of direct descendants nor the most persistent relationships with future coaches nor did they start their coaching careers the earliest. We conclude that, as a result of a simple hypothesized mechanism of influence, our method captures a rather general notion of position in the temporal influence network. Total influence is not dominated by apparent factors. This is unlike, for instance, in Jones et al. (36), where a time-dependent speaking measure is proposed to assess the positions of characters in networks of movie dialogs. Transferring that approach to the AFL, we would determine coaches’ influence from the temporal network of coaches “speaking” to future coaches they are currently coaching. Our experiments showed that, for most coaches, this results in a centrality value primarily determined by the number of seasons they have coached future coaches.

The current study extends the existing research on coaching trees, which to date has been primarily concerned with the question of whether success is inherited down (7, 8). It gives credit to a hypothetical influence of coaches on future coaches regardless of their explicit success. In this way, it contributes to the literature that supports the relevance of the coach–athlete association for coach development. However, it is noted that the used data do not provide qualitative interpretations and insights on the values and behaviors of coaches, which remains an unresolved issue (24). Moreover, the proposed model takes into account the timing and the order in which influence propagation within the coaching network takes place. Whereas when using methods that work with networks aggregated in time, the timing and order of relationships are lost. This applies in particular to approaches that rank nodes based on process over an aggregated network, such as PageRank (37) on a competition network of players (38), or “ball flow” across a players’ passing network (39). This loss persists even when methods take time into account within the aggregation process, as is the case with an aging weight (40–42).

### 4.1. Limitations

There are also limitations in this study. First, it is assumed that every coach is influenced by their former coaches, and coaching leads to independence from these influences. However, this assumption does not precisely capture individual differences between coaches and their relationships.

Second, the use of global parameters implies that coaches are susceptible to influence and that past influence decays in a homogeneous manner. This homogeneity helps prevent parameter explosion and overfitting in the model. However, the model could be extended to include different parameter classes that account for shared differences among groups of individuals.

Third, the validity of the model is challenging to verify due to the difficulty in measuring influence. The lack of an immediate unit of measurement further complicates the assessment of influence (43).

Fourth, the analysis of interpersonal influence and player development into coaches is limited to the AFL. The current data do not consider the influence of non-AFL coaches on AFL coaches, particularly those who had their playing careers in other leagues (such as *Chris Fagan*).^10^ Additionally, the data does not account for coaches who began their coaching careers in other leagues but later returned to the AFL as coaches (such as *Michael Nunan*).^11^ While these cases are rare in the AFL, defining the network boundaries when using this method may pose a more significant challenge in other sports.

## 5. Conclusion

We have presented a novel process of influence propagation and applied it to a temporal network of AFL coaching relationships, which we consider a proxy for direct influence. We found that our model identifies those coaches as influential who are highly reputable but that there is no single characteristic on which the influence value is dependent in isolation. This lack of dependence suggests that the influence value describes the overall position of the coach in the network and is not an artifact of the data. This study contributes to research on Australian Rules Football by examining characteristics of coaching and coach–future coach relationships in the AFL. It extends the limited amount of research on coaching trees and, in turn, also the emerging field of social network analysis in sports research. The proposed process has the potential to be applied not only to coaching trees in any sport but also to dynamic social relationships that may not be related to sport. A plausible direction for future research is to study the impact of alternative aggregations (rather than a mere summation of outgoing influences) on the nodes’ ranking.

## Data Availability

Publicly available datasets were analyzed in this study. These data can be found here: https://afltables.com/.

## Footnotes

^1^See, e.g., https://www.washingtonpost.com/graphics/2018/sports/nfl-coaching-trees-connecting-every-active-coach/, https://www.espn.com/nfl/story/_/page/greatestcoach130521/roots-nfl-coaching-trees-run-deep, https://www.espn.com/espn/feature/story/_/id/22235093/connecting-all-2018-nfl-head-coaches-bill-parcells-bill-belichick, http://graphics.wsj.com/nfl-coaches, Accessed 23 February 2021.

^2^https://afltables.com/, Accessed 23 June 2021.

^3^A list of sources is given at https://afltables.com/afl/notes.html, Accessed 23 June 2021.

^4^https://www.foxsports.com.au/afl/afl-2020-playing-coaches-return-to-afl-heath-shaw-players-also-coaching-in-games-coronavirus-financial-impact-gws/news-story/9e36509fe6fcbf8317eca99be0a2b89e, Accessed 1 March 2021.

^5^Accessed 23rd June 2021

^6^https://www.afl.com.au/hall-of-fame, Accessed 1 July 2021

^7^For coaches with the same amount of outgoing influence, the highest rank is chosen for both.

^8^https://www.afl.com.au/hall-of-fame/legends, Accessed 1 July 2021.

^9^https://www.afl.com.au/hall-of-fame/about, Accessed 1 July 2021.

^10^https://australianfootball.com/players/player/Chris%2BFagan/19774, Accessed 9 May 2023.

^11^https://australianfootball.com/players/player/Mick+Nunan/10482, https://australianfootball.com/coaches/coach/Michael%2BNunan/299, Accessed 9 February 2021.
